# Supplementary material for: Zeolite-confined subnanometric PtSn mimicking mortise-and-tenon joinery for catalytic propane dehydrogenation
Source: Nat Commun. 2022 May 17;13:2716. doi: 10.1038/s41467-022-30522-1 (PMC9114386; doi:10.1038/s41467-022-30522-1)
Supplement: Supplementary file 1 — Supplementary Information [file 41467_2022_30522_MOESM1_ESM.pdf]

Supplementary Information

Zeolite-Confined Subnanometric PtSn Mimicking Mortise-and-tenon Joinery for Catalytic Propane Dehydrogenation

Sicong Ma[1,2] and Zhi-Pan Liu[2,3]*

[1] Key Laboratory of Synthetic and Self-Assembly Chemistry for Organic Functional Molecules, Shanghai Institute of Organic Chemistry, Chinese Academy of Sciences, Shanghai 200032, China

[2] Shanghai Key Laboratory of Molecular Catalysis and Innovative Materials, Key Laboratory of Computational Physical Science, Department of Chemistry, Fudan University, Shanghai 200433, China

[3] Shanghai Qi Zhi Institution, Shanghai 200030, China

Corresponding Author: *zpliu@fudan.edu.cn

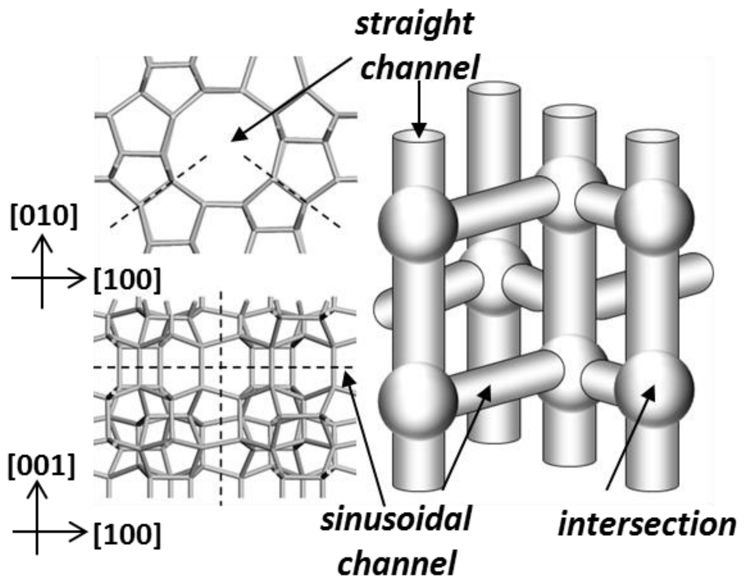

Supplementary Fig. 1 The structure of MFI-type zeolite.

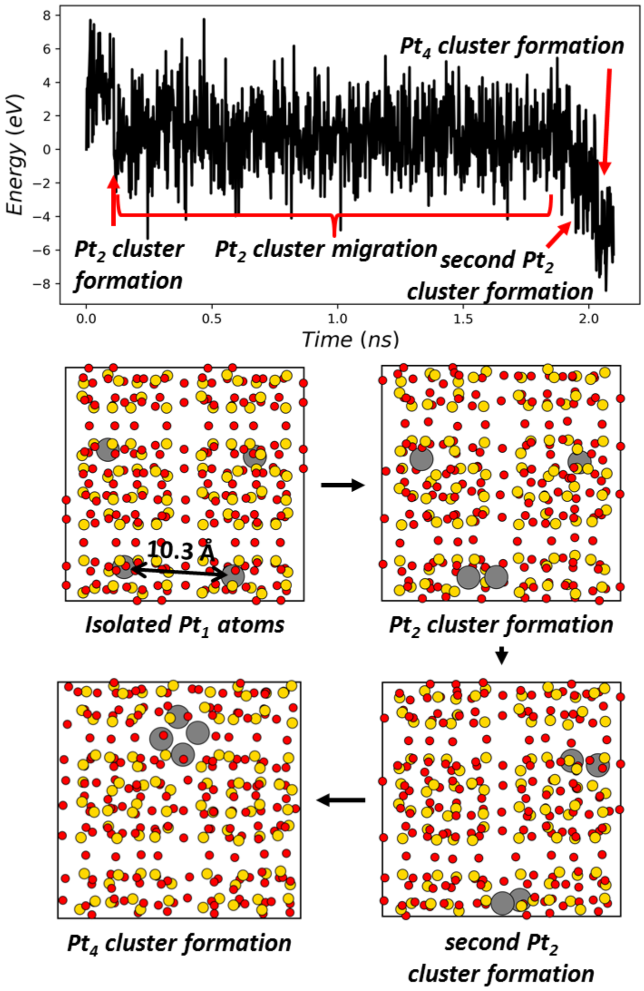

Supplementary Fig. 2 The energy variations and structure snapshots during MD simulation for the agglomeration of four isolated Pt_1 atoms to Pt_4 cluster within the MFI zeolite at 773 K. The initial distance between each two Pt_1 atoms is larger than 10 Å.

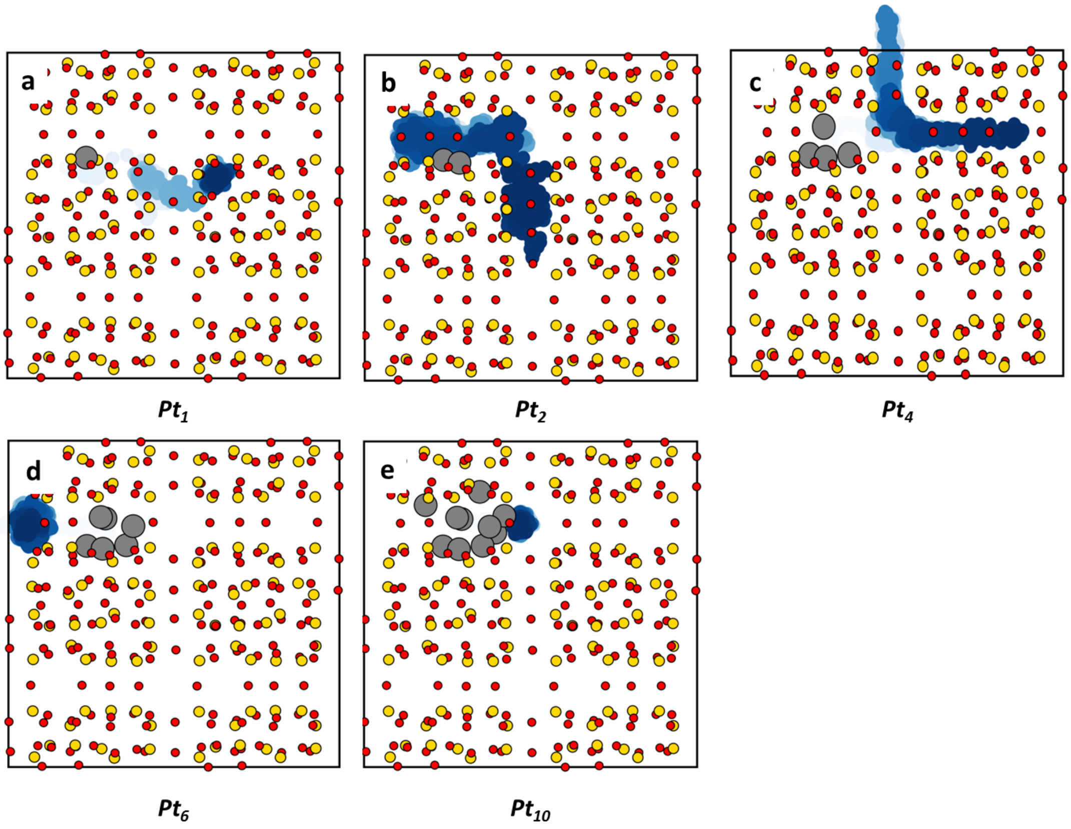

Supplementary Fig. 3 MD simulation trajectories of different Pt_x clusters within the MFI zeolite at 773 K, including (a) Pt_1@MFI, (b) Pt_2@MFI, (c) Pt_4@MFI, (d) Pt_6@MFI and (e) Pt_{10}@MFI. The structure models are depicted as the background with the clusters initially located at the sinusoidal channels of the zeolite. The position of the cluster in MD trajectory is given by the deep blue color dot and the color depth reflects the residence time. Gray, yellow and red balls represent the Pt, Si and O atoms, respectively.

The ultra-small Pt_x clusters with $x \leq 4$ show the wide distribution in MFI channels. These Pt_x clusters appear at not only sinusoidal channels but also straight channels (Supplementary Fig. 3a-c), indicating that the ultra-small Pt_x clusters can freely migrate in the MFI channels and would not be affected by the confinement effect of zeolite. Increasing the size of Pt_x clusters to $x \geq 6$, the more concentrated distributions are observed (Supplementary Fig. 3d-e). These Pt_x clusters migrate quickly from the initially sinusoidal channels to the intersections, and stay at the intersections without carrying out long-distance migration.

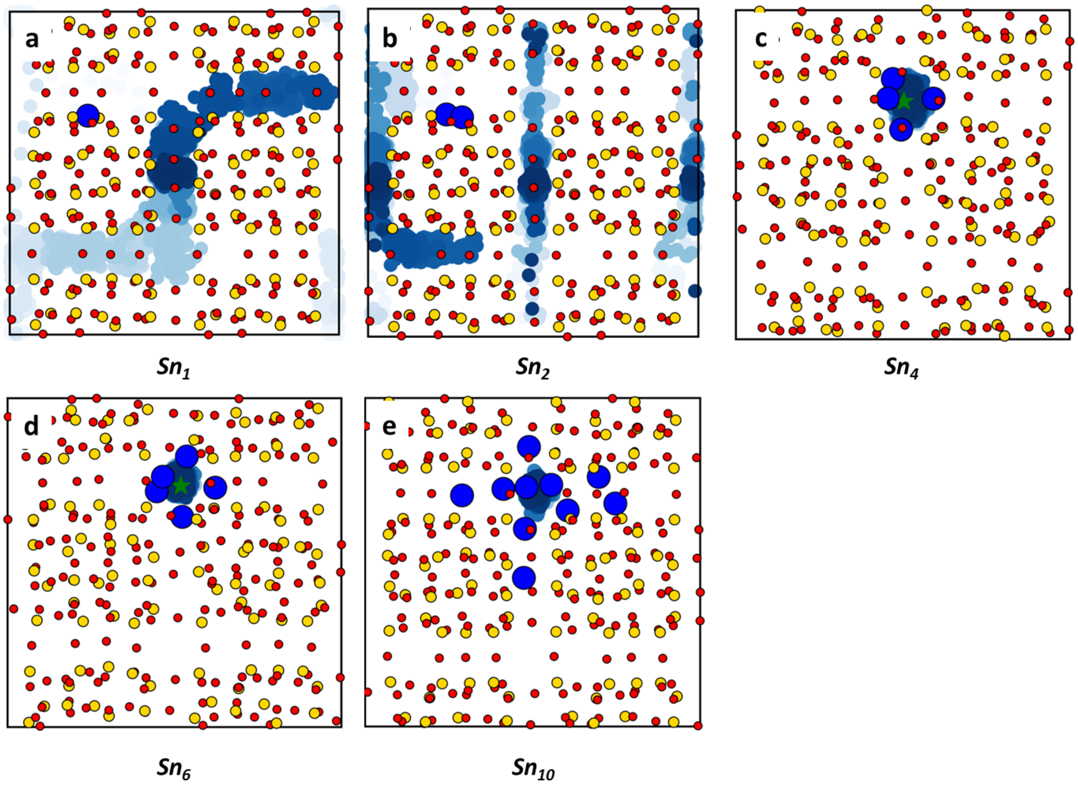

Supplementary Fig. 4 MD simulation trajectories of different Sn_x clusters within the MFI zeolite at 773 K, including (a) Sn_1@MFI, (b) Sn_2@MFI, (c) Sn_4@MFI, (d) Sn_6@MFI and (e) Sn_{10}@MFI. The structure models are depicted as the background with the clusters initially located at the sinusoidal channels of the zeolite. The position of the cluster in MD trajectory is given by the deep blue color dot and the color depth reflects the residence time. Light Blue, yellow and red balls represent the Sn, Si and O atoms, respectively.

The ultra-small Sn_x clusters with $x < 4$ show the wide distribution in MFI channels. These Sn_x clusters appear at not only sinusoidal channels but also straight channels (Supplementary Fig. 4a-b), indicating that the ultra-small Sn_x clusters can freely migrate in the MFI channels and would not be affected by the confinement effect of zeolite. Increasing the size of Sn_x clusters to $x \geq 4$, the more concentrated distributions are observed (Supplementary Fig. 4c-e). These Sn_x clusters migrate quickly from the initially sinusoidal channels to the intersections, and stay at the intersections without carrying out long-distance migration.

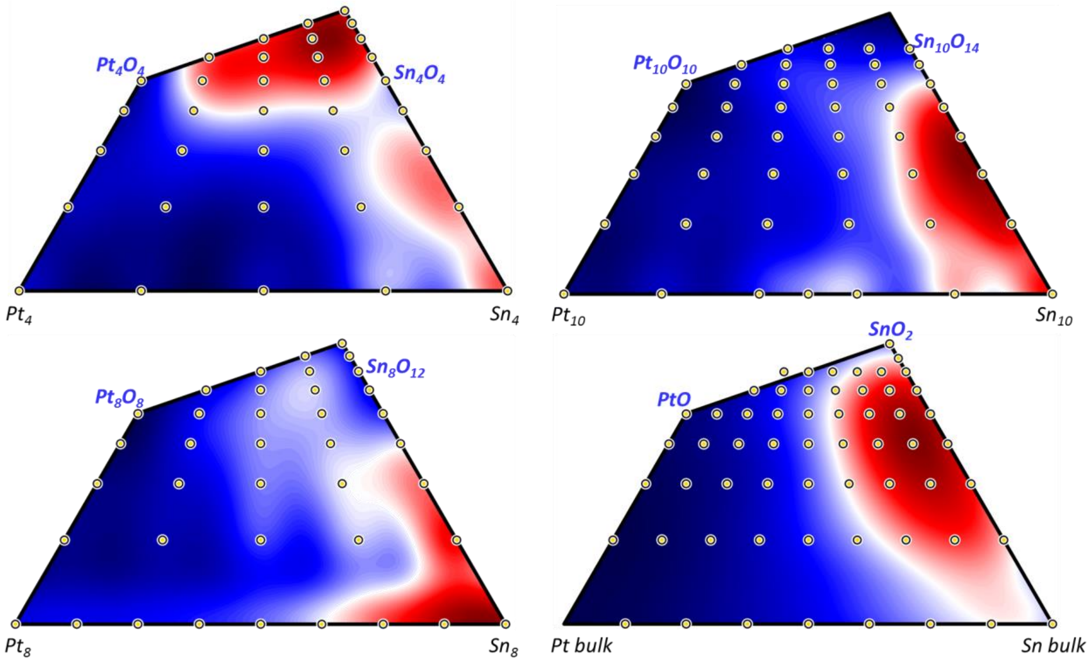

Supplementary Fig. 5 Ternary phase diagram for $Pt_xSn_yO_z$@MFI (x + y= 4, 8 and 10) clusters and bulk PtSnO under calcination condition.

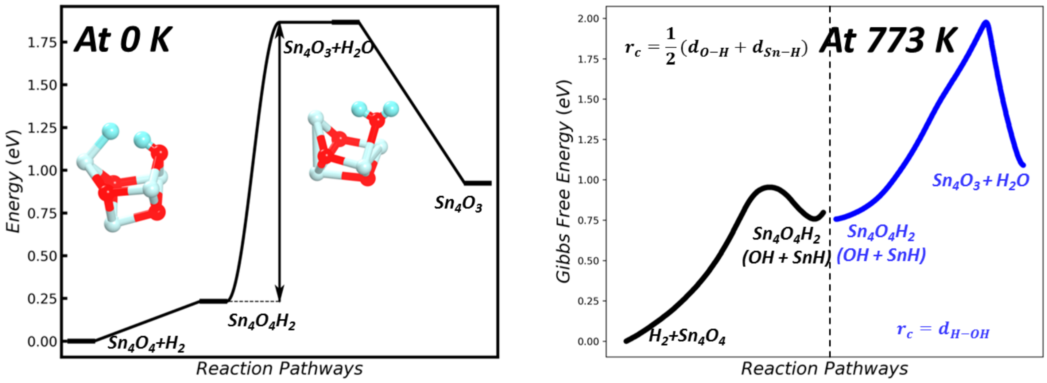

Supplementary Fig. 6 The Gibbs free energy profile at 0 K and 773 K for H_2 dissociated adsorption and OH/H coupling to H_2O on Sn_4O_4 cluster. The high-temperature Gibbs free energy is calculated based on MD simulation with umbrella sampling method.

The MD simulation with umbrella sampling method at 773 K was performed to calculate the free energy profile of Sn_4O_4 reduction by H_2. The Sn_4O_4 reduction process mainly involves two steps: H_2 dissociated adsorption to O-H and Sn-H and the OH/H coupling to H_2O. The reaction coordinates for H_2 adsorption and OH/H coupling are the distances of O-H and Sn-H and the distance between H and hydroxyl oxygen, respectively.

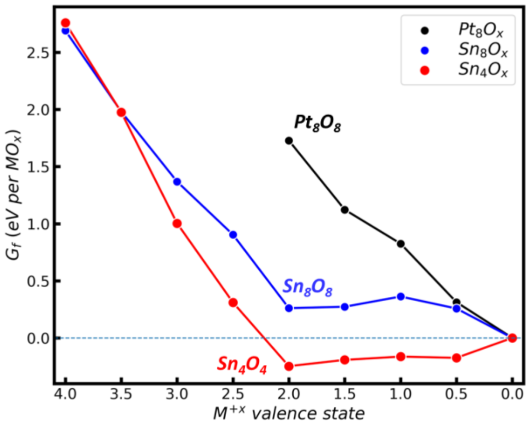

Supplementary Fig. 7 The G_f variations of SnO_x and PtO_x clusters during H_2 reduction process at 773 K.

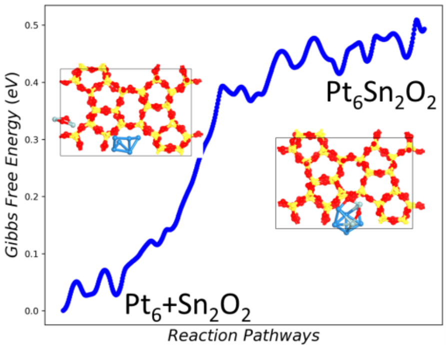

Supplementary Fig. 8 The Gibbs free energy profile for Sn_2O_2 and Pt_6 cluster agglomeration to $Pt_6Sn_2O_2$ at 773 K. The high-temperature Gibbs free energy is calculated based on MD simulation with umbrella sampling method. The distance between one of Sn atoms and one of Pt atoms is chosen as the reaction coordinate.

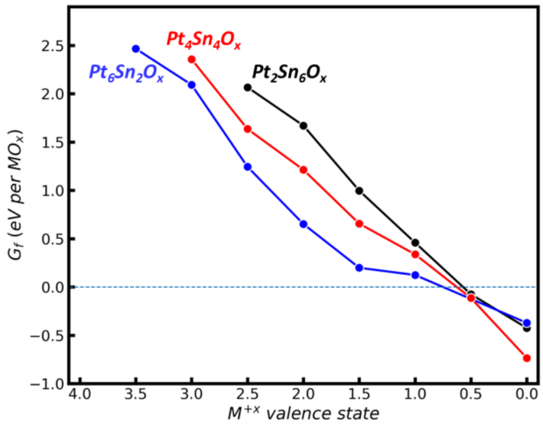

Supplementary Fig. 9 The G_f variation of $PtSnO_x$ clusters during H_2 reduction process at 773 K. The x axis represents the average metal oxidation state.

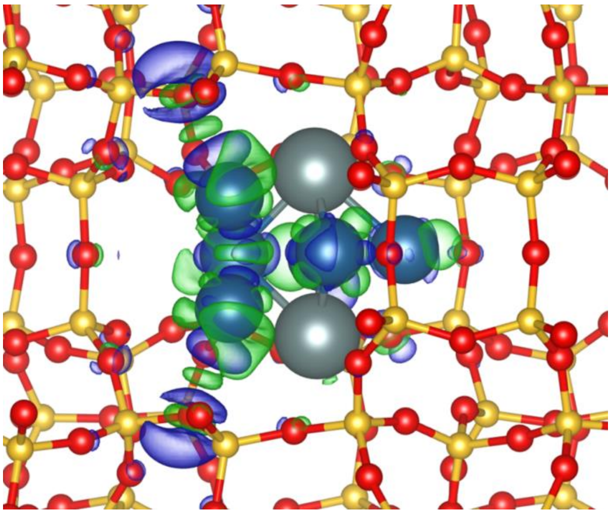

Supplementary Fig. 10 Charge density difference contour plot before and after the presence of Pt_6Sn_2 cluster. The green and blue colors indicate the increase and decrease in the electron density, respectively. The 3D isosurface value is set as 0.001 $e^-/Å^3$.

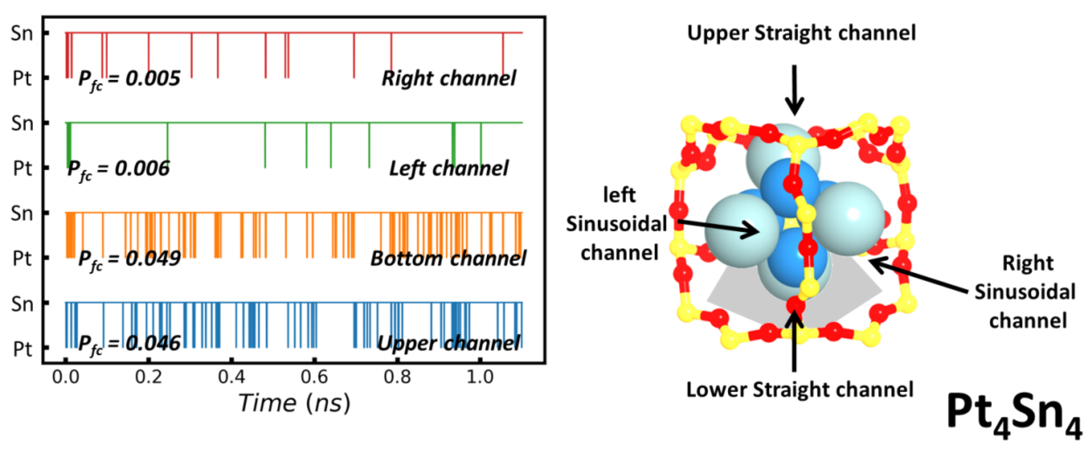

Supplementary Fig. 11 The MD trajectory of metal atom types towards the channel at 773 K for Pt_4Sn_4@MFI over 1 ns. The average probability of Pt atoms towards the channel is only 0.03, much lower than the theoretical concentration of Pt in cluster, i.e. 0.5.

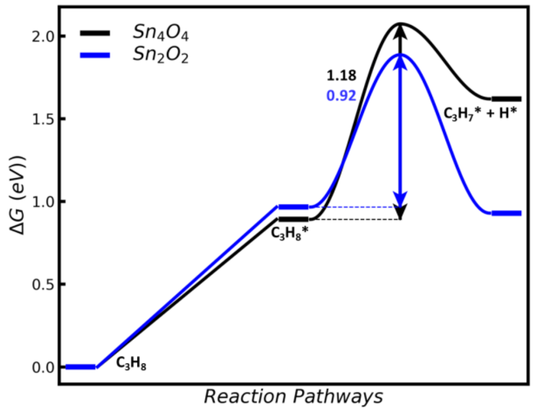

Supplementary Fig. 12 The Gibbs free energy profiles of propane activation on Sn_4O_4 and Sn_2O_2 clusters at 773 K calculated by PBE functional with D3 van der Waals correction.

The energy barriers of propane activation are 2.07 and 1.89 eV for Sn_4O_4 and Sn_2O_2 clusters, respectively. It is quite larger than that on PtSn clusters, pointing that the Sn_4O_4 and Sn_2O_2 clusters are inert for PDH reaction.

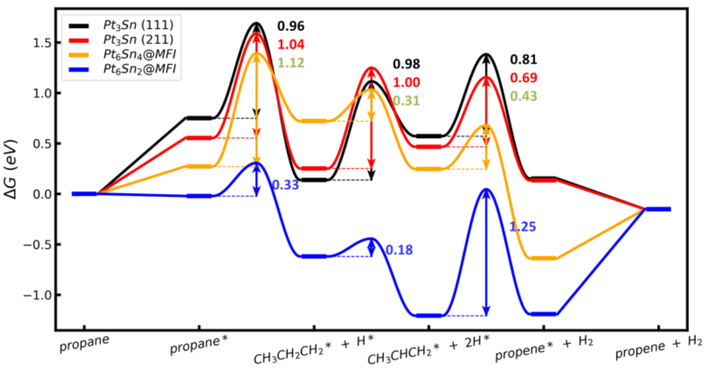

Supplementary Fig. 13 The Gibbs free energy profiles of PDH reaction on Pt_6Sn_2@MFI, Pt_6Sn_4@MFI, Pt_3Sn (111) and (211) surfaces at 773 K calculated by PBE functional with D3 van der Waals correction.

The total PDH reaction barriers for Pt_3Sn (111), Pt_3Sn (211), Pt_6Sn_4@MFI and Pt_6Sn_2@MFI are 1.71, 1.59, 1.39 and 1.25 eV, respectively.

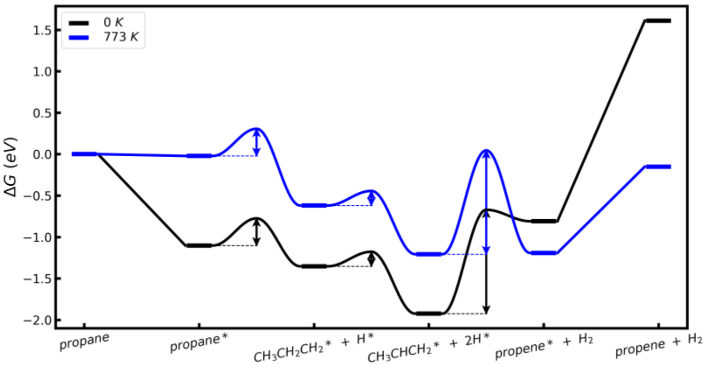

Supplementary Fig. 14 The Gibbs free energy profiles of PDH reaction on Pt_6Sn_2@MFI at 0 and 773 K.

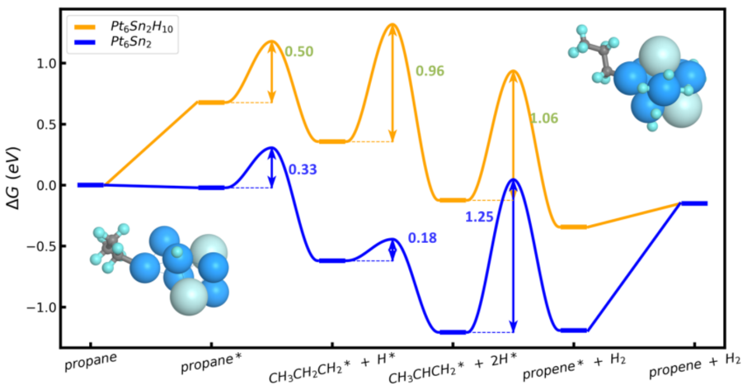

Supplementary Fig. 15 The Gibbs free energy profiles of PDH reaction on Pt_6Sn_2 clusters with and without high H coverage at 773 K calculated by PBE functional with D3 van der Waals correction.

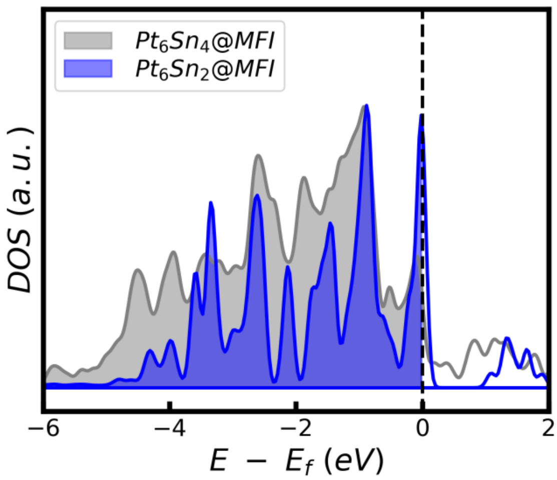

Supplementary Fig. 16 Projected density of states of Pt 5d orbitals for Pt_6Sn_4@MFI and Pt_6Sn_2@MFI. The Fermi level is set as energy zero.

The projected density of states (PDOS) of Pt 5d orbitals for Pt_6Sn_4@MFI and Pt_6Sn_2@MFI further show that the occupied states near the Fermi energy for Pt_6Sn_4@*MFI* have the smaller population than that for Pt_6Sn_2@*MFI*. It suggests that these Pt atoms on Pt_6Sn_4@*MFI* would form weaker covalent bonds with coming molecules, which inhabits the dissociation reactions (e.g. C_3H_8* -> C_3H_7* + H* and C_3H_7* -> C_3H_6* + H*; * represents the adsorption sites) but promotes the binding reaction and desorption (e.g. 2H* -> H_2 and C_3H_6* -> C_3H_6 + *). This is why the Gibbs free energy profile changes so dramatically with the increase of the number of Sn atoms from Pt_6Sn_2 to Pt_6Sn_4 in Supplementary Fig. 13.

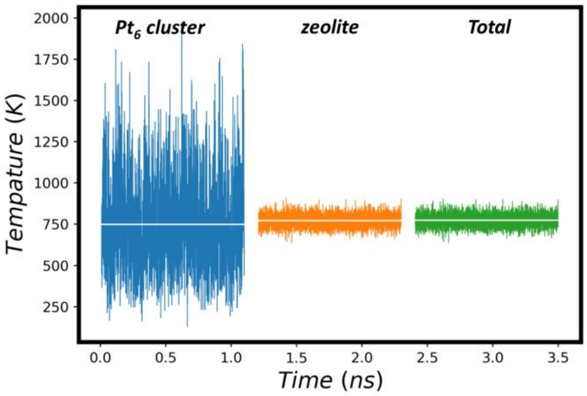

Supplementary Fig. 17 The temperature variation as a function of MD simulation time for different components of Pt_6@MFI at 773 K.

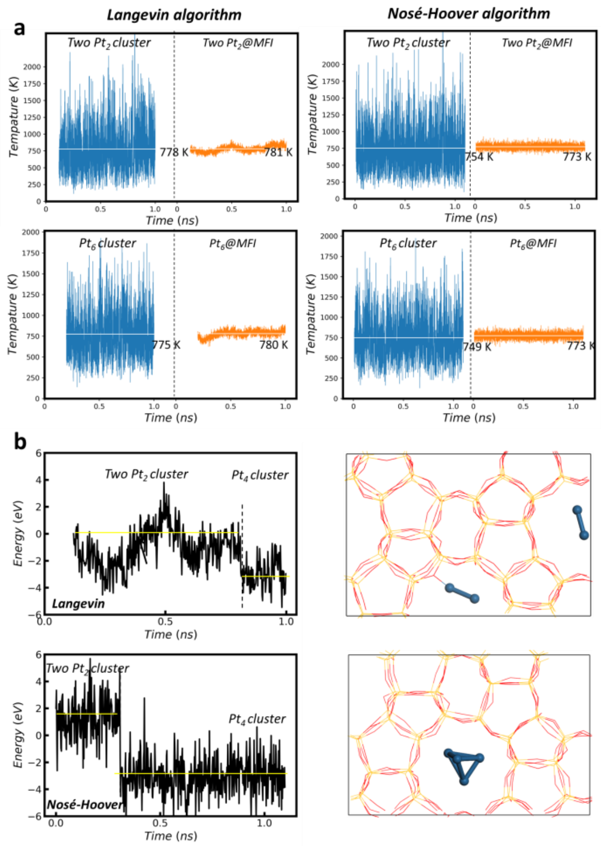

Supplementary Fig. 18 The comparison of MD simulation results between different thermostats. (a) The temperature and variation as a function of MD simulation time for Pt_n clusters within MFI zeolite at 773 K using Langevin and Nosé-Hoover thermostats. The average temperature for the Pt_n cluster is shown to compare with the overall temperature of the system. **(b)** The energy variation as a function of MD simulation time and structures for the agglomeration of two Pt_2 cluster to one Pt_4 cluster with different MD algorithms.

The Supplementary Fig. 18a compares the results for MD simulation of two different Pt_x clusters (Pt_2 and Pt_6) within MFI zeolite at 773 K using Langevin and Nosé-Hoover thermostats. It can be seen that the Nosé-Hoover thermostat, although has a better reserve the overall temperature (i.e. 773 K), the temperature gradient between the Pt_n subsystems and the zeolite is indeed larger, i.e. ~ 20 K. The Langevin thermostat can achieve the smaller temperature gradient between subsystems, but the overall temperature constancy is less accurate (i.e. ~780 K). It is important to note that the thermodynamics tendency for the growth of Pt_n cluster, which is the focus of the work, is not dependent on the MD thermostat, i.e. the same result being obtained for Langevin and Nosé-Hoover thermostat. As shown in the Supplementary Fig. 18b, we compare the trajectories for the Pt_2 cluster in zeolite by using two different thermostats. Both show that two Pt_2 cluster merges into one Pt_4 cluster within 1 ns. The final geometry for the Pt_4 cluster is identical from two MD trajectory, as shown in Supplementary Fig. 18b.

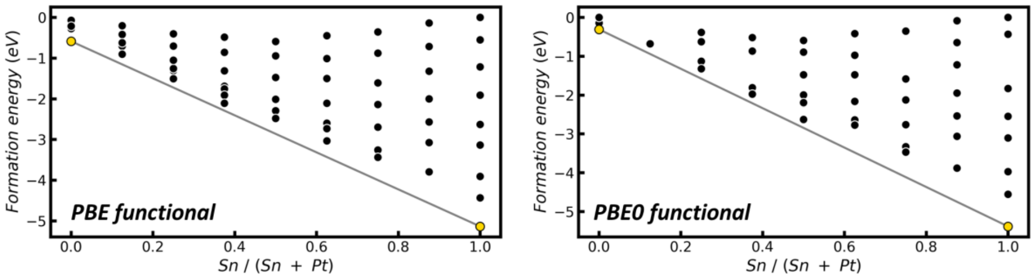

Supplementary Fig. 19 The thermodynamic convex hull for different bulk $Pt_xSn_yO_z$ compositions under O_2 condition calculated by PBE and PBE0 functionals.

Supplementary Table 1. The PDH reaction energy barrier and reaction rate constant at 773 K.

Pt_3Sn (111)	$E_{a,+}$ (eV)	$E_{a,-}$ (eV)	ΔG (eV)	k_+	k_-
* + C_3H_8 -> C_3H_8*	0.749	0.000	0.749	2.09E+08	1.61E+13
C_3H_8* + $ -> $CH_3CH_2CH_2$* + H$	0.942	1.553	-0.612	1.17E+07	1.20E+03
$CH_3CH_2CH_2$* + $ -> CH_3CHCH_2* + H$	0.977	0.543	0.434	6.86E+06	4.66E+09
2H$ -> H_2 + 2$	0.811	1.231	-0.421	8.32E+07	1.51E+05
CH_3CHCH_2* -> CH_3CHCH_2 + *	1.037	0.954	0.083	2.80E+06	9.71E+06
**Pt_6Sn_2@MFI**	**$E_{a,+}$**	**$E_{a,-}$**	**ΔG (eV)**	**k_+**	**k_-**
* + C_3H_8 -> C_3H_8*	0.000	0.022	-0.022	1.61E+13	1.17E+13
C_3H_8* + $ -> $CH_3CH_2CH_2$* + H$	0.327	0.926	-0.599	1.18E+11	1.48E+07
$CH_3CH_2CH_2$* + $ -> CH_3CHCH_2* + H$	0.176	0.763	-0.587	1.14E+12	1.70E+08
2H$ -> H_2 + 2$	1.252	1.237	0.016	1.10E+05	1.39E+05
CH_3CHCH_2* -> CH_3CHCH_2 + *	1.040	0.000	1.040	2.66E+06	1.61E+13

Computation for propene yield

Our calculated the PDH reaction rate is ~ 1.1 * 10^5 s^{-1} at 773 ^{K}. To estimate the propane in mol C3 per mol Pt s^{-1} to compare with experiment, we make the following derivation.

Considering the existence of Pt_xSn_y alloys with varied Pt:Sn ratio and particle sizes, we assume that the concentrate of Pt_6Sn_2 cluster is 0.1 ‰.

And the Pt atomic efficiency of Pt_6Sn_2 cluster is 1/6.

Therefore,

1.1 * 10^5 s^{-1} (Pt_6Sn_2) = 0.1 ‰ * 1/6 * 1.4 * 10^5 s^{-1} (total Pt) = 2.3 s^{-1} (total Pt) = 1.8 mol C3 per mol Pt s^{-1}

Supplementary Table 2. The screened zeolite candidates with the similar structural features with MFI-type zeolite for encapsulating subnanometric $PtSnO_x$ cluster.

code	rings	Includesphere[a]	Diffusesphere[b]
IMF	[10, 6, 5, 4]	7.34	[5.44, 1.84, 5.2]
ITH	[10, 9, 6, 5, 4]	6.72	[3.53, 5.13, 4.99]
ITR	[10, 9, 6, 5, 4]	6.36	[5.12, 5.12, 3.58]
MEL	[10, 8, 6, 5, 4]	7.72	[5.19, 5.19, 5.19]
MFI	[10, 6, 5, 4]	6.36	[4.7, 4.46, 4.46]
NES	[10, 6, 5, 4]	7.04	[5.07, 5.07, 2.06]
SFG	[10, 7, 6, 5, 4]	6.96	[4.98, 2.62, 5.38]
TER	[10, 6, 5, 4]	6.94	[5.16, 1.94, 4.74]
WEN	[10, 8, 6, 4]	5.53	[4.84, 4.84, 3.16]

[a] Maximum diameter of a sphere that can be included.

[b] Maximum diameter of a sphere that can be diffuse along a, b, c directions.

Supplementary Methods

Self-learning for NN potential construction

The neutral network (NN) potential is generated by iterative self-learning of the plane wave density functional theory (DFT) global potential energy surface (PES) dataset. At first, we need to prepare an initial global PES dataset which covers all the likely compositions of Pt-Sn-Si-O systems. Then the NN potential is generated using the method as introduced in our previous work (*J. Chem. Phys.* 2019, 151, 050901). It starts from generating a first-generation NN potential using the initial dataset which contains ~ 73,000 structures. This first-generation NN potential is then used to carry out long-time SSW/MD-NN simulation. A small additional dataset is thus obtained from the SSW/MD sampling trajectories, containing the structures on PES either randomly selected or exhibiting new atomic environment (e.g., out-of-bounds in structural descriptor, unrealistic energy/force/curvature). After calculating these additional data by DFT, they are added into the training dataset and the whole self-learning procedure returns back to the previous stage. Typically, after ∼100 iterations, a robust and accurate NN potential can be obtained with a compact training set that contains the most representative structures. It is worth noting that we would add a small amount of the structures that we are concerned about (e.g. $PtSnO_x$@MFI) to the dataset and then retrain to obtain the final NN potential function. The final Pt-Sn-Si-O training data set consists of 76,667 structures, which is openly accessible from the LASP Web site (see Web page link: *www.lasphub.com/supportings/Trainfile_PtSnSiO.tgz*). In final dataset, the atom number varies from 2 to 312 atoms per cell and has different Pt:Sn:Si:O ratios, e.g. Pt and Sn metals, PtSn alloys, $PtSnO_x$, $PtSiO_x$, $SnSiO_x$ and the composite of $PtSnO_x$ with SiO_2 zeolite). Among, 1889 $PtSnO_x$ structures are in cluster form and 2421 $PtSnO_x$ clusters are staying within SiO_2 zeolite. More detailed description of the data set in composition is listed in Supplementary Table 3.

Transition state search methods: DESW

The method operates two images starting from the initial and the final states, respectively, to walk in a stepwise manner toward each other until they meet. Once the pathway building is complete, we select the highest energy image from the chain and utilize the constrained Broyden dimer (CBD) method to locate the transition state exactly. The CBD method contains two independent modules, namely, the dimer rotation and the translation. The dimer rotation is to identify the reaction coordinate, an associated eigenvector of Hessian matrix with the negative eigenvalue, using a numerical finite difference method. Then the structure is translated gradually toward the TS along the reaction coordinate using a Quasi-Newton Broyden method. Finally, the identified transition states will be verified by further vibrational frequency analysis, which should have one and only one imaginary frequency along the reaction coordinate.

Supplementary Table 3. Structure information in the first principles global dataset that are utilized for fitting the global neural network potential. Listed data are the number of the structures in the global dataset, as distinguished by the chemical formula, the number of atoms per cell (N_{atom}), the type of structures (cluster, bulk, layer).

No.	Species	N_{atom}	cluster	layer	bulk	total	No.	Species	N_{atom}	cluster	layer	bulk	total
1	Pt12	12	0	0	4	4	441	O18-Sn7-Pt1	26	0	1	0	1
2	Pt16	16	1103	3	3804	4910	442	O18-Sn7-Pt2	27	0	1	0	1
3	Pt31	31	0	0	64	64	443	O18-Sn7-Pt5	30	0	0	2	2
4	Pt32	32	0	5	79	84	444	O18-Sn8	26	0	31	34	65
5	Sn1-Pt7	8	0	0	155	155	445	O18-Sn8-Pt1	27	0	91	176	267
6	Sn2	2	0	1	6	7	446	O18-Sn8-Pt4	30	0	0	2	2
7	Sn2-Pt6	8	0	0	74	74	447	O18-Sn9	27	0	39	52	91
8	Sn2-Pt10	12	0	0	6	6	448	O18-Sn9-Pt3	30	0	0	1	1
9	Sn2-Pt14	16	0	46	75	121	449	O18-Sn12	30	0	2	50	52
10	Sn3-Pt9	12	0	0	7	7	450	O19-Sn29-Pt30	78	0	124	0	124
11	Sn4	4	0	0	2	2	451	O20-Si10	30	0	1	302	303
12	Sn4-Pt8	12	0	0	14	14	452	O22-Sn6-Pt10	38	0	0	1	1
13	Sn4-Pt16	20	0	0	5	5	453	O22-Sn7-Pt9	38	0	0	3	3
14	Sn5-Pt3	8	0	13	65	78	454	O22-Sn8-Pt8	38	0	0	2	2
15	Sn5-Pt19	24	0	0	34	34	455	O22-Sn9-Pt7	38	0	0	5	5
16	Sn6-Pt2	8	0	9	53	62	456	O22-Sn10-Pt6	38	0	0	5	5
17	Sn6-Pt6	12	0	0	9	9	457	O22-Sn11-Pt5	38	0	0	1	1
18	Sn7-Pt1	8	0	3	70	73	458	O22-Sn12-Pt4	38	0	0	2	2
19	Sn8	8	1	0	1	2	459	O22-Sn13-Pt3	38	0	0	2	2
20	Sn8-Pt4	12	0	0	12	12	460	O22-Sn14-Pt2	38	0	0	2	2
21	Sn8-Pt12	20	0	0	12	12	461	O22-Sn16	38	0	0	128	128
22	Sn9-Pt3	12	0	0	9	9	462	O22-Sn21-Pt28	71	0	94	0	94
23	Sn10-Pt2	12	0	0	5	5	463	O22-Si11	33	0	10	316	326
24	Sn12	12	0	0	6	6	464	O24-Sn6-Pt10	40	0	0	1	1
25	Sn12-Pt8	20	0	0	2	2	465	O24-Sn10-Pt6	40	0	0	1	1
26	Sn14-Pt2	16	0	1	40	41	466	O24-Sn16	40	0	0	12	12
27	Sn16	16	505	3	3154	3662	467	O24-Si12	36	0	0	300	300
28	Sn16-Pt4	20	0	0	6	6	468	O24-Si12-Pt16	52	0	494	525	1019
29	Sn21-Pt3	24	0	0	90	90	469	O24-Si12-Sn1-Pt15	52	0	82	177	259
30	Sn21-Pt50	71	0	99	0	99	470	O24-Si12-Sn2-Pt14	52	0	65	112	177
31	Sn31	31	0	0	28	28	471	O24-Si12-Sn3-Pt13	52	0	38	66	104
32	Sn32	32	0	4	28	32	472	O24-Si12-Sn4-Pt12	52	0	30	75	105
33	Si1-Pt7	8	0	1	0	1	473	O24-Si12-Sn5-Pt11	52	0	28	75	103
34	Si1-Pt15	16	2	0	10	12	474	O24-Si12-Sn6-Pt10	52	0	29	109	138
35	Si1-Pt20	21	0	0	2	2	475	O24-Si12-Sn7-Pt9	52	0	19	44	63
36	Si1-Pt24	25	0	12	0	12	476	O24-Si12-Sn8-Pt8	52	0	18	55	73
37	Si1-Pt25	26	0	14	0	14	477	O24-Si12-Sn9-Pt7	52	0	27	59	86
38	Si1-Pt31	32	0	31	7	38	478	O24-Si12-Sn10-Pt6	52	0	22	59	81
39	Si2-Pt14	16	2	2	7	11	479	O24-Si12-Sn11-Pt5	52	0	9	43	52
40	Si2-Pt19	21	0	9	4	13	480	O24-Si12-Sn12-Pt4	52	0	15	42	57
41	Si2-Pt23	25	0	15	0	15	481	O24-Si12-Sn13-Pt3	52	0	10	18	28
42	Si2-Pt25	27	0	10	3	13	482	O24-Si12-Sn14-Pt2	52	0	2	9	11

43	Si2-Pt28	30	0	2	0	2	483	O24-Si12-Sn15-Pt1	52	0	2	0	2
44	Si2-Pt30	32	0	41	8	49	484	O25-Sn23-Pt30	78	0	78	0	78
45	Si2-Pt40	42	0	4	0	4	485	O25-Si12-Pt15	52	0	43	115	158
46	Si2-Pt48	50	0	0	6	6	486	O25-Si12-Sn1-Pt14	52	0	11	38	49
47	Si2-Pt62	64	0	2	26	28	487	O25-Si12-Sn2-Pt4	43	0	0	6	6
48	Si3-Pt5	8	0	1	0	1	488	O25-Si12-Sn2-Pt13	52	0	1	12	13
49	Si3-Pt13	16	0	0	6	6	489	O25-Si12-Sn3-Pt12	52	0	1	3	4
50	Si3-Pt18	21	0	3	5	8	490	O25-Si12-Sn4-Pt11	52	0	3	11	14
51	Si3-Pt25	28	0	25	0	25	491	O25-Si12-Sn5-Pt10	52	0	5	8	13
52	Si3-Pt28	31	0	3	1	4	492	O25-Si12-Sn6-Pt9	52	0	1	7	8
53	Si3-Pt29	32	0	12	2	14	493	O25-Si12-Sn7-Pt8	52	0	1	2	3
54	Si4-Pt12	16	1	0	5	6	494	O25-Si12-Sn8-Pt7	52	0	1	2	3
55	Si4-Pt17	21	0	1	0	1	495	O25-Si12-Sn9-Pt6	52	0	5	10	15
56	Si4-Pt25	29	0	9	1	10	496	O25-Si12-Sn10-Pt5	52	0	1	4	5
57	Si4-Pt28	32	0	63	15	78	497	O25-Si12-Sn11-Pt4	52	0	2	6	8
58	Si4-Pt38	42	0	3	4	7	498	O26-Si12-Pt14	52	0	38	69	107
59	Si4-Pt46	50	0	0	6	6	499	O26-Si12-Sn1-Pt13	52	0	9	23	32
60	Si4-Pt58	62	0	4	1	5	500	O26-Si12-Sn3-Pt4	45	0	0	4	4
61	Si4-Pt60	64	0	24	15	39	501	O26-Si12-Sn3-Pt11	52	0	1	0	1
62	Si4-Pt80	84	0	6	1	7	502	O26-Si12-Sn4-Pt10	52	0	2	5	7
63	Si4-Pt124	128	0	1	1	2	503	O26-Si12-Sn5-Pt9	52	0	3	11	14
64	Si5-Pt11	16	0	0	4	4	504	O26-Si12-Sn7-Pt7	52	0	6	2	8
65	Si5-Pt16	21	0	2	1	3	505	O26-Si12-Sn8-Pt6	52	0	2	5	7
66	Si5-Pt25	30	0	12	2	14	506	O26-Si12-Sn9-Pt5	52	0	2	5	7
67	Si5-Pt26	31	0	3	2	5	507	O26-Si12-Sn10-Pt4	52	0	1	6	7
68	Si5-Pt27	32	0	10	4	14	508	O26-Si12-Sn12-Pt2	52	0	2	1	3
69	Si5-Pt56	61	0	2	0	2	509	O27-Sn21-Pt23	71	0	50	0	50
70	Si6-Pt10	16	2	0	3	5	510	O27-Si12-Pt13	52	0	36	98	134
71	Si6-Pt15	21	0	4	0	4	511	O27-Si12-Sn1-Pt12	52	0	2	8	10
72	Si6-Pt24	30	0	13	2	15	512	O27-Si12-Sn2-Pt11	52	0	0	7	7
73	Si6-Pt25	31	0	13	0	13	513	O27-Si12-Sn3-Pt10	52	0	1	7	8
74	Si6-Pt26	32	0	72	6	78	514	O27-Si12-Sn4-Pt9	52	0	1	3	4
75	Si6-Pt36	42	0	10	3	13	515	O27-Si12-Sn7-Pt6	52	0	1	7	8
76	Si6-Pt50	56	0	1	7	8	516	O27-Si12-Sn10-Pt3	52	0	1	2	3
77	Si6-Pt56	62	0	1	3	4	517	O28-Si12-Pt12	52	0	10	39	49
78	Si6-Pt58	64	0	1	7	8	518	O28-Si12-Sn1-Pt11	52	0	5	35	40
79	Si6-Pt118	124	0	2	0	2	519	O28-Si12-Sn2-Pt10	52	0	1	5	6
80	Si7-Pt9	16	2	0	8	10	520	O28-Si12-Sn4-Pt8	52	0	4	4	8
81	Si7-Pt21	28	0	13	0	13	521	O29-Si12-Pt11	52	0	8	4	12
82	Si7-Pt25	32	0	28	8	36	522	O29-Si12-Sn1-Pt10	52	0	1	7	8
83	Si8-Pt13	21	0	0	1	1	523	O29-Si12-Sn2-Pt9	52	0	0	6	6
84	Si8-Pt22	30	0	0	1	1	524	O29-Si12-Sn3-Pt8	52	0	2	4	6
85	Si8-Pt24	32	0	40	8	48	525	O30-Sn23-Pt25	78	0	51	0	51
86	Si8-Pt34	42	0	1	0	1	526	O30-Si12-Pt10	52	0	5	6	11
87	Si8-Pt52	60	0	4	0	4	527	O30-Si12-Sn1-Pt9	52	0	0	1	1
88	Si8-Pt56	64	0	63	13	76	528	O30-Si12-Sn2-Pt8	52	0	1	0	1

89	Si8-Pt76	84	0	6	0	6	529	O30-Si12-Sn3-Pt7	52	0	1	4	5
90	Si8-Pt114	122	0	2	0	2	530	O31-Sn16-Pt4	51	0	92	0	92
91	Si8-Pt116	124	0	3	0	3	531	O31-Si12-Pt9	52	0	0	5	5
92	Si8-Pt120	128	0	11	9	20	532	O32-Pt16	48	33	70	17	120
93	Si9-Pt6	15	0	1	0	1	533	O32-Sn1-Pt15	48	0	1	0	1
94	Si9-Pt7	16	0	0	2	2	534	O32-Sn2-Pt14	48	0	1	1	2
95	Si10-Pt6	16	0	0	4	4	535	O32-Sn5-Pt11	48	0	1	0	1
96	Si10-Pt22	32	0	46	12	58	536	O32-Sn6-Pt10	48	0	2	0	2
97	Si10-Pt32	42	0	1	1	2	537	O32-Sn7-Pt9	48	0	0	1	1
98	Si10-Pt51	61	0	2	0	2	538	O32-Sn16-Pt4	52	0	111	1	112
99	Si10-Pt112	122	0	3	0	3	539	O32-Sn16-Pt16	64	0	159	1	160
100	Si11-Pt4	15	1	0	0	1	540	O32-Sn21-Pt18	71	0	97	0	97
101	Si11-Pt5	16	0	0	1	1	541	O32-Sn31-Pt8	71	0	99	0	99
102	Si11-Pt21	32	0	2	0	2	542	O32-Si12-Pt8	52	0	0	6	6
103	Si12-Pt4	16	1	1	8	10	543	O33-Sn16-Pt5	54	0	119	0	119
104	Si12-Pt19	31	0	1	1	2	544	O33-Sn16-Pt16	65	0	93	0	93
105	Si12-Pt20	32	0	34	20	54	545	O38-Sn23-Pt17	78	0	83	0	83
106	Si12-Pt30	42	0	3	1	4	546	O38-Sn32-Pt8	78	0	90	0	90
107	Si12-Pt48	60	0	2	5	7	547	O45-Sn22-Pt38	105	0	79	0	79
108	Si12-Pt50	62	0	0	3	3	548	O48-Si24-Pt30	102	0	440	443	883
109	Si12-Pt52	64	0	59	29	88	549	O48-Si24-Pt32	104	0	48	130	178
110	Si12-Pt72	84	0	14	3	17	550	O48-Si24-Sn1-Pt29	102	0	56	90	146
111	Si12-Pt108	120	0	2	0	2	551	O48-Si24-Sn1-Pt31	104	0	1	23	24
112	Si12-Pt112	124	0	3	0	3	552	O48-Si24-Sn2-Pt28	102	0	62	61	123
113	Si12-Pt116	128	0	9	1	10	553	O48-Si24-Sn2-Pt30	104	0	5	24	29
114	Si13-Pt2	15	1	0	0	1	554	O48-Si24-Sn3-Pt27	102	0	26	44	70
115	Si13-Pt3	16	1	0	3	4	555	O48-Si24-Sn3-Pt29	104	0	8	15	23
116	Si14	14	0	0	8	8	556	O48-Si24-Sn4-Pt26	102	0	24	42	66
117	Si14-Pt1	15	1	0	1	2	557	O48-Si24-Sn4-Pt28	104	0	0	12	12
118	Si14-Pt2	16	0	2	16	18	558	O48-Si24-Sn5-Pt25	102	0	12	16	28
119	Si14-Pt18	32	0	31	1	32	559	O48-Si24-Sn5-Pt27	104	0	3	2	5
120	Si14-Pt28	42	0	0	3	3	560	O48-Si24-Sn6-Pt24	102	0	17	20	37
121	Si14-Pt42	56	0	0	1	1	561	O48-Si24-Sn6-Pt26	104	0	4	10	14
122	Si14-Pt46	60	0	1	0	1	562	O48-Si24-Sn7-Pt23	102	0	21	31	52
123	Si14-Pt48	62	0	4	0	4	563	O48-Si24-Sn7-Pt25	104	0	0	5	5
124	Si14-Pt50	64	0	0	6	6	564	O48-Si24-Sn8-Pt22	102	0	14	20	34
125	Si15	15	125	0	15	140	565	O48-Si24-Sn8-Pt24	104	0	0	4	4
126	Si15-Pt1	16	1	0	6	7	566	O48-Si24-Sn9-Pt21	102	0	12	28	40
127	Si16	16	781	93	2921	3795	567	O48-Si24-Sn9-Pt23	104	0	1	4	5
128	Si16-Pt16	32	0	2	0	2	568	O48-Si24-Sn10-Pt20	102	0	17	21	38
129	Si16-Pt26	42	0	2	0	2	569	O48-Si24-Sn10-Pt22	104	0	2	14	16
130	Si16-Pt48	64	0	26	15	41	570	O48-Si24-Sn11-Pt19	102	0	10	13	23
131	Si16-Pt68	84	0	6	0	6	571	O48-Si24-Sn11-Pt21	104	0	2	4	6
132	Si16-Pt104	120	0	9	0	9	572	O48-Si24-Sn12-Pt18	102	0	6	8	14
133	Si16-Pt108	124	0	1	0	1	573	O48-Si24-Sn12-Pt20	104	0	2	13	15
134	Si16-Pt112	128	0	19	20	39	574	O48-Si24-Sn13-Pt17	102	0	9	9	18

135	Si16-Pt152	168	0	0	1	1	575	O48-Si24-Sn14-Pt16	102	0	5	15	20
136	Si17	17	0	5	6	11	576	O48-Si24-Sn14-Pt18	104	0	2	2	4
137	Si18-Pt12	30	0	2	0	2	577	O48-Si24-Sn15-Pt15	102	0	8	8	16
138	Si18-Pt14	32	0	10	1	11	578	O48-Si24-Sn16-Pt14	102	0	14	24	38
139	Si20-Pt10	30	0	4	0	4	579	O48-Si24-Sn16-Pt16	104	0	1	4	5
140	Si20-Pt12	32	0	28	15	43	580	O48-Si24-Sn17-Pt13	102	0	8	29	37
141	Si20-Pt44	64	0	34	25	59	581	O48-Si24-Sn17-Pt15	104	0	0	7	7
142	Si20-Pt64	84	0	3	1	4	582	O48-Si24-Sn18-Pt12	102	0	13	36	49
143	Si20-Pt96	116	0	2	0	2	583	O48-Si24-Sn18-Pt14	104	0	0	5	5
144	Si20-Pt100	120	0	1	0	1	584	O48-Si24-Sn19-Pt11	102	0	12	22	34
145	Si20-Pt102	122	0	0	1	1	585	O48-Si24-Sn19-Pt13	104	0	1	7	8
146	Si20-Pt104	124	0	4	0	4	586	O48-Si24-Sn20-Pt10	102	0	8	22	30
147	Si20-Pt108	128	0	8	1	9	587	O48-Si24-Sn20-Pt12	104	0	0	7	7
148	Si22-Pt8	30	0	4	0	4	588	O48-Si24-Sn21-Pt9	102	0	7	24	31
149	Si22-Pt10	32	0	33	9	42	589	O48-Si24-Sn21-Pt11	104	0	1	1	2
150	Si22-Pt20	42	0	1	1	2	590	O48-Si24-Sn22-Pt8	102	0	1	7	8
151	Si22-Pt40	62	0	0	2	2	591	O48-Si24-Sn22-Pt10	104	0	1	8	9
152	Si23-Pt8	31	0	2	0	2	592	O48-Si24-Sn23-Pt7	102	0	1	3	4
153	Si24-Pt6	30	0	6	1	7	593	O48-Si24-Sn23-Pt9	104	0	0	2	2
154	Si24-Pt8	32	0	50	15	65	594	O48-Si24-Sn24-Pt6	102	0	2	3	5
155	Si24-Pt40	64	0	21	26	47	595	O48-Si24-Sn25-Pt7	104	0	0	2	2
156	Si24-Pt60	84	0	8	2	10	596	O49-Si24-Pt29	102	0	21	25	46
157	Si24-Pt96	120	0	1	2	3	597	O49-Si24-Pt31	104	0	1	29	30
158	Si24-Pt100	124	0	3	0	3	598	O49-Si24-Sn1-Pt28	102	0	1	5	6
159	Si24-Pt104	128	0	39	28	67	599	O49-Si24-Sn1-Pt30	104	0	0	4	4
160	Si24-Pt144	168	0	1	0	1	600	O49-Si24-Sn2-Pt27	102	0	2	0	2
161	Si26-Pt4	30	0	0	1	1	601	O49-Si24-Sn3-Pt26	102	0	3	3	6
162	Si26-Pt6	32	0	48	24	72	602	O49-Si24-Sn4-Pt25	102	0	0	3	3
163	Si26-Pt96	122	0	4	1	5	603	O49-Si24-Sn5-Pt24	102	0	0	2	2
164	Si28-Pt2	30	0	2	0	2	604	O49-Si24-Sn6-Pt25	104	0	0	1	1
165	Si28-Pt4	32	0	69	19	88	605	O49-Si24-Sn7-Pt22	102	0	6	1	7
166	Si28-Pt36	64	0	17	12	29	606	O49-Si24-Sn8-Pt21	102	0	0	3	3
167	Si28-Pt56	84	0	1	0	1	607	O49-Si24-Sn8-Pt23	104	0	0	1	1
168	Si28-Pt92	120	0	0	4	4	608	O49-Si24-Sn10-Pt19	102	0	1	6	7
169	Si28-Pt96	124	0	4	1	5	609	O49-Si24-Sn11-Pt20	104	0	0	4	4
170	Si28-Pt100	128	0	6	0	6	610	O49-Si24-Sn12-Pt17	102	0	7	1	8
171	Si30	30	0	0	5	5	611	O49-Si24-Sn12-Pt19	104	0	0	2	2
172	Si30-Pt2	32	0	49	6	55	612	O49-Si24-Sn13-Pt16	102	0	1	0	1
173	Si30-Pt4	34	0	3	0	3	613	O49-Si24-Sn14-Pt15	102	0	0	3	3
174	Si31	31	0	0	6	6	614	O49-Si24-Sn16-Pt13	102	0	4	10	14
175	Si32	32	0	0	27	27	615	O49-Si24-Sn16-Pt15	104	0	1	5	6
176	Si32-Pt32	64	0	1	1	2	616	O49-Si24-Sn18-Pt11	102	0	2	0	2
177	Si32-Pt52	84	0	8	0	8	617	O49-Si24-Sn19-Pt10	102	0	0	4	4
178	Si32-Pt88	120	0	0	3	3	618	O49-Si24-Sn20-Pt9	102	0	2	7	9
179	Si32-Pt96	128	0	14	10	24	619	O49-Si24-Sn21-Pt8	102	0	1	0	1
180	Si32-Pt136	168	0	0	1	1	620	O50-Si24-Pt28	102	0	26	32	58

181	Si36-Pt28	64	0	28	9	37	621	O50-Si24-Pt30	104	0	3	15	18
182	Si36-Pt84	120	0	1	0	1	622	O50-Si24-Sn1-Pt27	102	0	2	6	8
183	Si40-Pt24	64	0	24	8	32	623	O50-Si24-Sn1-Pt29	104	0	1	0	1
184	Si40-Pt80	120	0	1	0	1	624	O50-Si24-Sn2-Pt26	102	0	0	1	1
185	Si40-Pt84	124	0	5	0	5	625	O50-Si24-Sn2-Pt28	104	0	0	1	1
186	Si40-Pt88	128	0	20	14	34	626	O50-Si24-Sn3-Pt25	102	0	2	2	4
187	Si40-Pt128	168	0	0	1	1	627	O50-Si24-Sn3-Pt27	104	0	0	1	1
188	Si42-Pt18	60	0	1	0	1	628	O50-Si24-Sn5-Pt23	102	0	2	0	2
189	Si44-Pt20	64	0	43	16	59	629	O50-Si24-Sn6-Pt22	102	0	0	1	1
190	Si44-Pt80	124	0	1	0	1	630	O50-Si24-Sn7-Pt21	102	0	1	1	2
191	Si48-Pt12	60	0	3	0	3	631	O50-Si24-Sn8-Pt22	104	0	0	1	1
192	Si48-Pt16	64	0	48	38	86	632	O50-Si24-Sn9-Pt19	102	0	1	0	1
193	Si48-Pt80	128	0	20	19	39	633	O50-Si24-Sn10-Pt18	102	0	2	1	3
194	Si52-Pt8	60	0	6	1	7	634	O50-Si24-Sn11-Pt17	102	0	0	1	1
195	Si52-Pt12	64	0	39	15	54	635	O50-Si24-Sn11-Pt19	104	0	0	2	2
196	Si52-Pt76	128	0	1	0	1	636	O50-Si24-Sn12-Pt16	102	0	1	0	1
197	Si56-Pt4	60	0	5	0	5	637	O50-Si24-Sn12-Pt18	104	0	0	4	4
198	Si56-Pt8	64	0	64	31	95	638	O50-Si24-Sn13-Pt15	102	0	0	7	7
199	Si56-Pt72	128	0	6	5	11	639	O50-Si24-Sn14-Pt14	102	0	1	5	6
200	Si60-Pt4	64	0	34	7	41	640	O50-Si24-Sn15-Pt13	102	0	2	3	5
201	Si64-Pt64	128	0	2	7	9	641	O50-Si24-Sn17-Pt11	102	0	1	0	1
202	Si64-Pt104	168	0	3	0	3	642	O50-Si24-Sn18-Pt10	102	0	2	2	4
203	Si72-Pt56	128	0	3	13	16	643	O50-Si24-Sn18-Pt12	104	0	0	2	2
204	Si80-Pt48	128	0	14	8	22	644	O50-Si24-Sn19-Pt9	102	0	1	0	1
205	Si88-Pt32	120	0	1	0	1	645	O50-Si24-Sn19-Pt11	104	0	0	2	2
206	Si88-Pt40	128	0	20	27	47	646	O50-Si24-Sn20-Pt8	102	0	2	5	7
207	Si96-Pt24	120	0	4	3	7	647	O50-Si24-Sn20-Pt10	104	0	0	1	1
208	Si96-Pt32	128	0	16	24	40	648	O51-Si24-Pt27	102	0	20	50	70
209	Si100-Pt28	128	0	7	0	7	649	O51-Si24-Pt29	104	0	2	13	15
210	Si104-Pt16	120	0	0	1	1	650	O51-Si24-Sn1-Pt26	102	0	1	5	6
211	Si104-Pt24	128	0	22	26	48	651	O51-Si24-Sn2-Pt25	102	0	0	3	3
212	Si108-Pt16	124	0	1	0	1	652	O51-Si24-Sn3-Pt24	102	0	2	2	4
213	Si112-Pt8	120	0	4	3	7	653	O51-Si24-Sn4-Pt23	102	0	2	2	4
214	Si112-Pt16	128	0	13	30	43	654	O51-Si24-Sn5-Pt24	104	0	0	4	4
215	Si120-Pt8	128	0	19	10	29	655	O51-Si24-Sn6-Pt21	102	0	2	1	3
216	O1-Pt12	13	0	176	0	176	656	O51-Si24-Sn7-Pt20	102	0	0	1	1
217	O1-Sn1-Pt8	10	0	11	83	94	657	O51-Si24-Sn7-Pt22	104	0	0	4	4
218	O1-Sn1-Pt11	13	0	5	0	5	658	O51-Si24-Sn9-Pt18	102	0	0	1	1
219	O1-Sn2-Pt10	13	0	6	0	6	659	O51-Si24-Sn10-Pt17	102	0	1	1	2
220	O1-Sn3-Pt9	13	0	3	0	3	660	O51-Si24-Sn12-Pt15	102	0	0	1	1
221	O1-Sn3-Pt13	17	0	0	1	1	661	O51-Si24-Sn12-Pt17	104	0	0	1	1
222	O1-Sn4-Pt8	13	0	4	0	4	662	O51-Si24-Sn14-Pt13	102	0	2	10	12
223	O1-Sn5-Pt7	13	0	2	0	2	663	O51-Si24-Sn15-Pt12	102	0	0	5	5
224	O1-Sn5-Pt11	17	0	0	1	1	664	O51-Si24-Sn16-Pt11	102	0	1	7	8
225	O1-Sn6-Pt6	13	0	9	0	9	665	O51-Si24-Sn17-Pt10	102	0	0	1	1
226	O1-Sn7-Pt5	13	0	4	0	4	666	O51-Si24-Sn18-Pt9	102	0	0	2	2

227	O1-Sn8-Pt4	13	0	2	0	2	667	O51-Si24-Sn19-Pt8	102	0	2	1	3
228	O1-Sn8-Pt8	17	0	0	3	3	668	O51-Si24-Sn19-Pt10	104	0	0	2	2
229	O1-Sn9-Pt3	13	0	5	0	5	669	O51-Si24-Sn20-Pt7	102	0	4	1	5
230	O1-Sn9-Pt7	17	0	0	1	1	670	O51-Si24-Sn20-Pt9	104	0	0	5	5
231	O1-Sn10-Pt2	13	0	2	0	2	671	O51-Si24-Sn21-Pt6	102	0	0	4	4
232	O1-Sn10-Pt6	17	0	0	2	2	672	O52-Si24-Pt26	102	0	23	30	53
233	O1-Sn11-Pt1	13	0	2	0	2	673	O52-Si24-Pt28	104	0	0	7	7
234	O1-Sn11-Pt5	17	0	0	1	1	674	O52-Si24-Sn1-Pt25	102	0	3	4	7
235	O1-Sn12	13	0	70	0	70	675	O52-Si24-Sn2-Pt24	102	0	0	3	3
236	O1-Sn16	17	0	0	40	40	676	O52-Si24-Sn2-Pt26	104	0	0	5	5
237	O2-Pt10	12	0	161	0	161	677	O52-Si24-Sn3-Pt23	102	0	1	0	1
238	O2-Sn1-Pt8	11	0	5	70	75	678	O52-Si24-Sn3-Pt25	104	0	0	2	2
239	O2-Sn1-Pt9	12	0	4	0	4	679	O52-Si24-Sn4-Pt22	102	0	0	3	3
240	O2-Sn2-Pt8	12	0	6	0	6	680	O52-Si24-Sn5-Pt21	102	0	1	1	2
241	O2-Sn2-Pt16	20	0	0	27	27	681	O52-Si24-Sn6-Pt20	102	0	1	1	2
242	O2-Sn3-Pt7	12	0	3	0	3	682	O52-Si24-Sn6-Pt22	104	0	0	1	1
243	O2-Sn4-Pt6	12	0	7	0	7	683	O52-Si24-Sn12-Pt16	104	0	0	3	3
244	O2-Sn5-Pt5	12	0	4	0	4	684	O52-Si24-Sn16-Pt10	102	0	2	4	6
245	O2-Sn6-Pt4	12	0	2	0	2	685	O52-Si24-Sn18-Pt10	104	0	0	1	1
246	O2-Sn8-Pt2	12	0	2	0	2	686	O53-Si24-Pt25	102	0	13	33	46
247	O2-Sn10	12	0	78	0	78	687	O53-Si24-Pt27	104	0	3	6	9
248	O2-Sn13-Pt3	18	0	0	1	1	688	O53-Si24-Sn1-Pt24	102	0	1	1	2
249	O2-Sn16	18	0	0	39	39	689	O53-Si24-Sn1-Pt26	104	0	1	2	3
250	O3-Pt16	19	24	0	0	24	690	O53-Si24-Sn2-Pt23	102	0	1	1	2
251	O4	4	0	94	0	94	691	O53-Si24-Sn3-Pt22	102	0	1	0	1
252	O4-Pt8	12	0	0	4626	4626	692	O53-Si24-Sn3-Pt24	104	0	1	5	6
253	O4-Pt27	31	0	0	117	117	693	O53-Si24-Sn4-Pt21	102	0	0	2	2
254	O4-Sn1-Pt7	12	0	0	161	161	694	O53-Si24-Sn7-Pt18	102	0	1	0	1
255	O4-Sn1-Pt26	31	0	0	1	1	695	O53-Si24-Sn8-Pt17	102	0	0	5	5
256	O4-Sn2-Pt6	12	0	0	193	193	696	O53-Si24-Sn9-Pt16	102	0	1	8	9
257	O4-Sn2-Pt16	22	0	1	33	34	697	O53-Si24-Sn18-Pt7	102	0	1	0	1
258	O4-Sn2-Pt25	31	0	0	1	1	698	O54-Si24-Pt24	102	0	11	34	45
259	O4-Sn3-Pt5	12	0	0	169	169	699	O54-Si24-Pt26	104	0	0	10	10
260	O4-Sn3-Pt24	31	0	0	1	1	700	O54-Si24-Sn1-Pt23	102	0	3	2	5
261	O4-Sn4-Pt4	12	0	0	144	144	701	O54-Si24-Sn2-Pt24	104	0	1	3	4
262	O4-Sn4-Pt23	31	0	0	1	1	702	O54-Si24-Sn3-Pt21	102	0	2	3	5
263	O4-Sn5-Pt3	12	0	0	109	109	703	O54-Si24-Sn3-Pt23	104	0	0	1	1
264	O4-Sn5-Pt22	31	0	0	2	2	704	O54-Si24-Sn4-Pt20	102	0	0	4	4
265	O4-Sn6-Pt2	12	0	0	69	69	705	O54-Si24-Sn5-Pt19	102	0	0	3	3
266	O4-Sn6-Pt10	20	0	0	1	1	706	O54-Si24-Sn8-Pt16	102	0	0	1	1
267	O4-Sn6-Pt21	31	0	0	2	2	707	O54-Si24-Sn19-Pt5	102	0	7	0	7
268	O4-Sn7-Pt1	12	0	0	38	38	708	O55-Sn22-Pt28	105	0	49	0	49
269	O4-Sn7-Pt20	31	0	0	2	2	709	O55-Si24-Pt23	102	0	13	27	40
270	O4-Sn8	12	0	0	2080	2080	710	O55-Si24-Pt25	104	0	1	15	16
271	O4-Sn8-Pt19	31	0	0	2	2	711	O55-Si24-Sn1-Pt22	102	0	1	0	1
272	O4-Sn9-Pt18	31	0	0	3	3	712	O55-Si24-Sn1-Pt24	104	0	0	3	3

273	O4-Sn10-Pt6	20	0	0	2	2
274	O4-Sn10-Pt17	31	0	1	1	2
275	O4-Sn11-Pt5	20	0	0	1	1
276	O4-Sn11-Pt16	31	0	0	1	1
277	O4-Sn12-Pt15	31	0	1	2	3
278	O4-Sn13-Pt14	31	0	0	3	3
279	O4-Sn14-Pt13	31	0	0	1	1
280	O4-Sn15-Pt12	31	0	2	0	2
281	O4-Sn16	20	0	1	34	35
282	O4-Sn16-Pt11	31	0	0	2	2
283	O4-Sn19-Pt8	31	0	0	3	3
284	O4-Sn20-Pt7	31	0	0	1	1
285	O4-Sn22-Pt5	31	0	0	3	3
286	O4-Sn27	31	0	11	46	57
287	O5-Sn4-Pt5	14	0	22	70	92
288	O6-Pt4	10	0	66	218	284
289	O6-Pt26	32	0	0	100	100
290	O6-Sn1-Pt3	10	0	4	54	58
291	O6-Sn1-Pt8	15	0	11	73	84
292	O6-Sn1-Pt25	32	0	0	1	1
293	O6-Sn2-Pt2	10	0	5	97	102
294	O6-Sn3-Pt1	10	0	2	79	81
295	O6-Sn3-Pt23	32	0	0	5	5
296	O6-Sn4	10	0	28	1962	1990
297	O6-Sn4-Pt22	32	0	0	1	1
298	O6-Sn5-Pt21	32	0	0	1	1
299	O6-Sn6-Pt20	32	0	0	1	1
300	O6-Sn7-Pt19	32	0	0	1	1
301	O6-Sn8	14	32	0	0	32
302	O6-Sn8-Pt18	32	0	0	2	2
303	O6-Sn9-Pt17	32	0	0	2	2
304	O6-Sn11-Pt15	32	0	0	2	2
305	O6-Sn13-Pt13	32	0	0	1	1
306	O6-Sn14-Pt12	32	0	0	1	1
307	O6-Sn17-Pt9	32	0	0	2	2
308	O6-Sn19-Pt7	32	0	0	1	1
309	O6-Sn26	32	0	7	38	45
310	O7-Pt8	15	0	0	4567	4567
311	O7-Sn1-Pt7	15	0	0	174	174
312	O7-Sn2-Pt6	15	0	0	186	186
313	O7-Sn3-Pt5	15	0	0	187	187
314	O7-Sn4-Pt4	15	0	0	153	153
315	O7-Sn5-Pt3	15	0	0	147	147
316	O7-Sn5-Pt11	23	0	1	1	2
317	O7-Sn6-Pt2	15	0	0	109	109
318	O7-Sn6-Pt10	23	0	0	2	2
713	O55-Si24-Sn2-Pt21	102	0	0	3	3
714	O55-Si24-Sn2-Pt23	104	0	1	5	6
715	O55-Si24-Sn3-Pt20	102	0	1	0	1
716	O55-Si24-Sn8-Pt15	102	0	0	1	1
717	O56-Si24-Pt22	102	0	6	9	15
718	O56-Si24-Sn4-Pt18	102	0	0	2	2
719	O57-Si24-Pt21	102	0	4	11	15
720	O57-Si24-Pt23	104	0	1	2	3
721	O57-Si24-Sn2-Pt19	102	0	1	0	1
722	O57-Si24-Sn3-Pt20	104	0	0	3	3
723	O57-Si24-Sn4-Pt17	102	0	0	1	1
724	O57-Si24-Sn9-Pt12	102	0	0	5	5
725	O58-Si24-Pt20	102	0	6	6	12
726	O58-Si24-Sn1-Pt19	102	0	1	6	7
727	O58-Si24-Sn3-Pt17	102	0	1	0	1
728	O59-Si24-Pt21	104	0	1	2	3
729	O65-Sn22-Pt18	105	0	121	0	121
730	O65-Sn32-Pt8	105	0	120	0	120
731	O192-Si96-Pt4	292	0	0	23	23
732	O192-Si96-Pt6	294	0	0	1	1
733	O192-Si96-Pt8	296	0	0	1	1
734	O192-Si96-Pt9	297	0	0	69	69
735	O192-Si96-Pt10	298	0	0	13	13
736	O192-Si96-Sn1-Pt3	292	0	0	1	1
737	O192-Si96-Sn1-Pt7	296	0	0	1	1
738	O192-Si96-Sn1-Pt8	297	0	0	136	136
739	O192-Si96-Sn2-Pt2	292	0	0	2	2
740	O192-Si96-Sn2-Pt6	296	0	0	1	1
741	O192-Si96-Sn2-Pt7	297	0	0	157	157
742	O192-Si96-Sn2-Pt8	298	0	0	1	1
743	O192-Si96-Sn3-Pt1	292	0	0	14	14
744	O192-Si96-Sn3-Pt5	296	0	0	2	2
745	O192-Si96-Sn3-Pt6	297	0	0	33	33
746	O192-Si96-Sn4	292	0	4	9	13
747	O192-Si96-Sn4-Pt4	296	0	0	2	2
748	O192-Si96-Sn4-Pt5	297	0	0	46	46
749	O192-Si96-Sn5-Pt3	296	0	0	2	2
750	O192-Si96-Sn5-Pt4	297	0	0	65	65
751	O192-Si96-Sn6-Pt2	296	0	0	2	2
752	O192-Si96-Sn6-Pt3	297	0	0	29	29
753	O192-Si96-Sn6-Pt4	298	0	0	1	1
754	O192-Si96-Sn7-Pt1	296	0	0	2	2
755	O192-Si96-Sn7-Pt2	297	0	0	28	28
756	O192-Si96-Sn8	296	0	0	2	2
757	O192-Si96-Sn8-Pt2	298	0	0	2	2
758	O192-Si96-Sn10	298	0	0	2	2

319	O7-Sn7-Pt1	15	0	0	42	42	759	O193-Si96-Pt1	290	0	0	1	1
320	O7-Sn8	15	0	0	2932	2932	760	O193-Si96-Pt4	293	0	0	1	1
321	O7-Sn10-Pt6	23	0	0	1	1	761	O193-Si96-Pt6	295	0	0	1	1
322	O7-Sn12-Pt4	23	0	1	2	3	762	O193-Si96-Pt8	297	0	0	63	63
323	O7-Sn14-Pt2	23	0	0	2	2	763	O193-Si96-Sn1-Pt3	293	0	0	2	2
324	O7-Sn16	23	0	4	43	47	764	O193-Si96-Sn1-Pt7	297	0	0	65	65
325	O8-Pt6	14	0	19	173	192	765	O193-Si96-Sn2-Pt2	293	0	0	2	2
326	O8-Pt8	16	42	5	5732	5779	766	O193-Si96-Sn2-Pt6	297	0	0	113	113
327	O8-Pt16	24	26	0	0	26	767	O193-Si96-Sn3-Pt1	293	0	2	0	2
328	O8-Sn1-Pt5	14	0	2	10	12	768	O193-Si96-Sn4	293	0	0	2	2
329	O8-Sn1-Pt7	16	0	1	213	214	769	O194-Si96-Pt2	292	0	0	15	15
330	O8-Sn2-Pt4	14	0	2	6	8	770	O194-Si96-Pt4	294	0	0	3	3
331	O8-Sn2-Pt6	16	0	0	216	216	771	O194-Si96-Pt6	296	0	0	2	2
332	O8-Sn3-Pt3	14	0	0	10	10	772	O194-Si96-Pt7	297	0	0	49	49
333	O8-Sn3-Pt5	16	0	0	228	228	773	O194-Si96-Pt8	298	0	0	1	1
334	O8-Sn4-Pt2	14	0	1	6	7	774	O194-Si96-Pt10	300	0	0	7	7
335	O8-Sn4-Pt4	16	0	0	200	200	775	O194-Si96-Sn1-Pt3	294	0	0	1	1
336	O8-Sn4-Pt5	17	0	13	78	91	776	O194-Si96-Sn1-Pt6	297	0	0	14	14
337	O8-Sn5-Pt1	14	0	0	2	2	777	O194-Si96-Sn2-Pt2	294	0	0	2	2
338	O8-Sn5-Pt3	16	0	0	191	191	778	O194-Si96-Sn2-Pt6	298	0	0	5	5
339	O8-Sn6	14	0	9	65	74	779	O194-Si96-Sn2-Pt8	300	0	0	1	1
340	O8-Sn6-Pt2	16	0	0	157	157	780	O194-Si96-Sn3-Pt1	294	0	0	2	2
341	O8-Sn7-Pt1	16	0	0	73	73	781	O194-Si96-Sn4	294	0	0	2	2
342	O8-Sn8	16	0	0	4239	4239	782	O194-Si96-Sn4-Pt4	298	0	0	2	2
343	O8-Sn59-Pt38	105	0	89	0	89	783	O194-Si96-Sn4-Pt6	300	0	0	1	1
344	O10-Pt6	16	0	90	140	230	784	O194-Si96-Sn6-Pt4	300	0	0	1	1
345	O10-Sn1-Pt5	16	0	6	7	13	785	O194-Si96-Sn8	298	0	0	2	2
346	O10-Sn2-Pt4	16	0	4	5	9	786	O194-Si96-Sn8-Pt2	300	0	0	8	8
347	O10-Sn3-Pt3	16	0	7	4	11	787	O194-Si96-Sn10	300	0	0	1	1
348	O10-Sn4-Pt2	16	0	4	3	7	788	O195-Si96-Pt4	295	0	0	1	1
349	O10-Sn5-Pt1	16	0	0	3	3	789	O195-Si96-Sn1-Pt3	295	0	0	1	1
350	O10-Sn6	16	0	48	65	113	790	O195-Si96-Sn2-Pt2	295	0	0	1	1
351	O10-Sn7-Pt9	26	0	0	2	2	791	O195-Si96-Sn3-Pt1	295	0	0	2	2
352	O10-Sn8-Pt8	26	0	0	1	1	792	O196-Si96-Pt4	296	0	0	23	23
353	O10-Sn8-Pt10	28	0	2	58	60	793	O196-Si96-Pt6	298	0	0	1	1
354	O10-Sn9-Pt7	26	0	0	2	2	794	O196-Si96-Pt8	300	0	0	1	1
355	O10-Sn12-Pt4	26	0	0	1	1	795	O196-Si96-Pt10	302	0	0	4	4
356	O10-Sn13-Pt3	26	0	1	1	2	796	O196-Si96-Sn1-Pt3	296	0	0	1	1
357	O10-Sn15-Pt1	26	0	0	1	1	797	O196-Si96-Sn2	294	0	1	9	10
358	O10-Sn16	26	0	3	40	43	798	O196-Si96-Sn2-Pt2	296	0	0	1	1
359	O11	11	0	478	146	624	799	O196-Si96-Sn2-Pt6	300	0	0	1	1
360	O12-Sn3-Pt5	20	0	0	4	4	800	O196-Si96-Sn2-Pt8	302	0	0	2	2
361	O12-Sn4-Pt4	20	0	2	0	2	801	O196-Si96-Sn3-Pt1	296	0	0	1	1
362	O12-Sn5-Pt3	20	0	0	1	1	802	O196-Si96-Sn4	296	0	2	15	17
363	O12-Sn6-Pt2	20	0	1	1	2	803	O196-Si96-Sn4-Pt4	300	0	0	2	2
364	O12-Sn8	20	63	34	27	124	804	O196-Si96-Sn4-Pt6	302	0	0	1	1

365	O12-Sn9-Pt7	28	0	0	2	2	805	O196-Si96-Sn4-Pt8	304	0	0	5	5
366	O12-Sn13-Pt3	28	0	0	1	1	806	O196-Si96-Sn4-Pt10	306	0	0	5	5
367	O12-Sn14-Pt2	28	0	0	1	1	807	O196-Si96-Sn6-Pt2	300	0	0	2	2
368	O12-Sn15-Pt1	28	0	0	1	1	808	O196-Si96-Sn6-Pt4	302	0	0	1	1
369	O12-Sn16	28	0	3	32	35	809	O196-Si96-Sn8	300	0	9	14	23
370	O12-Si6	18	0	1368	11582	12950	810	O196-Si96-Sn8-Pt2	302	0	0	1	1
371	O14-Pt7	21	0	54	138	192	811	O196-Si96-Sn10	302	0	0	1	1
372	O14-Sn1-Pt6	21	0	2	3	5	812	O197-Si96-Sn1-Pt3	297	0	0	2	2
373	O14-Sn2-Pt5	21	0	7	6	13	813	O197-Si96-Sn3-Pt1	297	0	0	2	2
374	O14-Sn3-Pt4	21	0	1	3	4	814	O197-Si96-Sn4	297	0	0	1	1
375	O14-Sn3-Pt5	22	0	1	2	3	815	O198-Si96-Pt6	300	0	0	1	1
376	O14-Sn4-Pt3	21	0	3	3	6	816	O198-Si96-Pt8	302	0	0	2	2
377	O14-Sn4-Pt4	22	0	0	1	1	817	O198-Si96-Pt10	304	0	0	7	7
378	O14-Sn4-Pt12	30	0	0	1	1	818	O198-Si96-Sn2-Pt2	298	0	0	1	1
379	O14-Sn5-Pt2	21	0	0	2	2	819	O198-Si96-Sn2-Pt6	302	0	0	1	1
380	O14-Sn5-Pt3	22	0	0	2	2	820	O198-Si96-Sn2-Pt8	304	0	0	4	4
381	O14-Sn6-Pt1	21	0	0	1	1	821	O198-Si96-Sn3-Pt1	298	0	0	1	1
382	O14-Sn6-Pt2	22	0	0	2	2	822	O198-Si96-Sn4	298	0	0	2	2
383	O14-Sn7	21	0	28	52	80	823	O198-Si96-Sn4-Pt4	302	0	0	1	1
384	O14-Sn7-Pt1	22	0	0	1	1	824	O198-Si96-Sn4-Pt6	304	0	0	2	2
385	O14-Sn8	22	0	1	44	45	825	O198-Si96-Sn6-Pt2	302	0	0	18	18
386	O14-Sn9-Pt7	30	0	1	1	2	826	O198-Si96-Sn6-Pt4	304	0	0	1	1
387	O14-Sn12-Pt4	30	0	2	0	2	827	O198-Si96-Sn8	302	0	0	1	1
388	O14-Sn16	30	0	15	18	33	828	O198-Si96-Sn8-Pt2	304	0	0	1	1
389	O15-Sn6-Pt2	23	0	0	5	5	829	O198-Si96-Sn10	304	0	0	2	2
390	O15-Sn7-Pt1	23	0	0	1	1	830	O199-Si96-Sn3-Pt1	299	0	0	1	1
391	O15-Sn8	23	0	1	41	42	831	O199-Si96-Sn4	299	0	0	1	1
392	O16-Pt16	32	28	0	24	52	832	O200-Si96-Pt8	304	0	0	1	1
393	O16-Sn3-Pt5	24	0	1	0	1	833	O200-Si96-Pt10	306	0	0	4	4
394	O16-Sn4-Pt4	24	0	0	1	1	834	O200-Si96-Sn2-Pt6	304	0	0	1	1
395	O16-Sn4-Pt12	32	0	0	3	3	835	O200-Si96-Sn2-Pt8	306	0	0	1	1
396	O16-Sn5-Pt3	24	0	1	0	1	836	O200-Si96-Sn4	300	0	0	1	1
397	O16-Sn5-Pt11	32	0	1	1	2	837	O200-Si96-Sn4-Pt4	304	0	0	2	2
398	O16-Sn6-Pt2	24	0	1	0	1	838	O200-Si96-Sn6-Pt2	304	0	0	2	2
399	O16-Sn6-Pt10	32	0	1	0	1	839	O200-Si96-Sn6-Pt4	306	0	0	1	1
400	O16-Sn7-Pt1	24	0	0	1	1	840	O200-Si96-Sn8	304	0	0	2	2
401	O16-Sn7-Pt9	32	0	1	1	2	841	O200-Si96-Sn10	306	0	0	2	2
402	O16-Sn8	24	32	34	43	109	842	O202-Si96-Pt10	308	0	0	7	7
403	O16-Sn8-Pt1	25	0	69	76	145	843	O202-Si96-Sn2-Pt6	306	0	0	1	1
404	O16-Sn8-Pt8	32	0	2	1	3	844	O202-Si96-Sn2-Pt8	308	0	0	2	2
405	O16-Sn8-Pt10	34	0	1	51	52	845	O202-Si96-Sn4-Pt4	306	0	0	1	1
406	O16-Sn9-Pt7	32	0	2	0	2	846	O202-Si96-Sn4-Pt6	308	0	0	2	2
407	O16-Sn10-Pt6	32	0	2	0	2	847	O202-Si96-Sn6-Pt2	306	0	0	1	1
408	O16-Sn11-Pt5	32	0	3	0	3	848	O202-Si96-Sn6-Pt4	308	0	0	2	2
409	O16-Sn12-Pt4	32	0	1	0	1	849	O202-Si96-Sn8	306	0	0	22	22
410	O16-Sn14-Pt2	32	0	1	0	1	850	O202-Si96-Sn8-Pt2	308	0	0	1	1

411	O16-Sn16	32	0	79	13	92
412	O18-Pt7	25	0	66	116	182
413	O18-Pt8	26	0	50	86	136
414	O18-Pt9	27	0	61	110	171
415	O18-Pt12	30	0	2	133	135
416	O18-Sn1-Pt6	25	0	2	6	8
417	O18-Sn1-Pt7	26	0	3	5	8
418	O18-Sn1-Pt8	27	0	93	154	247
419	O18-Sn1-Pt11	30	0	0	6	6
420	O18-Sn2-Pt5	25	0	3	2	5
421	O18-Sn2-Pt6	26	0	1	4	5
422	O18-Sn2-Pt7	27	0	165	112	277
423	O18-Sn2-Pt10	30	0	0	8	8
424	O18-Sn3-Pt4	25	0	3	10	13
425	O18-Sn3-Pt5	26	0	3	5	8
426	O18-Sn3-Pt6	27	0	3	3	6
427	O18-Sn3-Pt9	30	0	0	3	3
428	O18-Sn4-Pt3	25	0	3	2	5
429	O18-Sn4-Pt4	26	0	2	2	4
430	O18-Sn4-Pt5	27	0	113	153	266
431	O18-Sn4-Pt8	30	0	0	2	2
432	O18-Sn5-Pt2	25	0	2	2	4
433	O18-Sn5-Pt3	26	0	1	4	5
434	O18-Sn5-Pt4	27	0	2	4	6
435	O18-Sn5-Pt7	30	0	0	3	3
436	O18-Sn6-Pt1	25	0	0	1	1
437	O18-Sn6-Pt2	26	0	2	1	3
438	O18-Sn6-Pt3	27	0	115	197	312
439	O18-Sn6-Pt6	30	0	1	1	2
440	O18-Sn7	25	0	31	50	81
851	O202-Si96-Sn10	308	0	0	2	2
852	O204-Si96-Sn2-Pt8	310	0	0	2	2
853	O204-Si96-Sn4-Pt4	308	0	0	2	2
854	O204-Si96-Sn4-Pt6	310	0	0	2	2
855	O204-Si96-Sn6-Pt2	308	0	0	2	2
856	O204-Si96-Sn6-Pt4	310	0	0	2	2
857	O204-Si96-Sn8	308	0	0	1	1
858	O204-Si96-Sn8-Pt2	310	0	0	2	2
859	O204-Si96-Sn10	310	0	0	2	2
860	O206-Si96-Sn4-Pt6	312	0	0	2	2
861	O206-Si96-Sn6-Pt2	310	0	0	2	2
862	O206-Si96-Sn6-Pt4	312	0	0	2	2
863	O206-Si96-Sn8	310	0	0	2	2
864	O206-Si96-Sn8-Pt2	312	0	0	1	1
865	O206-Si96-Sn10	312	0	0	1	1
866	O208-Si96-Sn8	312	0	0	2	2
**867**	**total**	**--**	**2810**	**10461**	**63396**	**76667**

Supplementary Table 4. Benchmark of G-NN and DFT formation Gibbs free energy (G_f) of $PtSnO_x$ clusters in MFI zeolite. The Gibbs free formation energy of $Pt_xSn_yO_z$@MFI (ΔG_f) is with respect to the energy of Pt_8@MFI, Sn_8@MFI and O_2 under calcination conditions.

Compositions	E_{NN} *(eV)*	E_{DFT} *(eV)*	ΔE_{DFT-NN} *(meV/atom)*	$G_{f,NN}$	$G_{f,DFT}$	$\Delta G_{f,DFT-NN}$ (eV per MO_x formula unit)
Pt0Sn8O0	-2302.883	-2302.932	-0.167	0.000	0.000	0.000
Pt0Sn8O10	-2376.566	-2376.395	0.560	-2.025	-1.997	0.028
Pt0Sn8O12	-2388.879	-2388.817	0.199	-2.127	-2.113	0.014
Pt0Sn8O14	-2400.156	-2400.050	0.342	-2.099	-2.080	0.019
Pt0Sn8O16	-2410.656	-2410.508	0.475	-1.975	-1.950	0.025
Pt0Sn8O2	-2316.852	-2317.012	-0.538	-0.309	-0.323	-0.014
Pt0Sn8O4	-2332.301	-2332.313	-0.039	-0.803	-0.798	0.005
Pt0Sn8O6	-2349.244	-2349.165	0.260	-1.484	-1.468	0.016
Pt0Sn8O8	-2365.514	-2365.402	0.370	-2.080	-2.060	0.020
Pt1Sn7O0	-2305.260	-2305.369	-0.368	-0.186	-0.200	-0.014
Pt2Sn6O0	-2307.587	-2307.585	0.008	-0.365	-0.372	-0.007
Pt2Sn6O10	-2375.237	-2375.350	-0.371	-1.635	-1.657	-0.022
Pt2Sn6O12	-2384.620	-2384.704	-0.275	-1.371	-1.389	-0.018
Pt2Sn6O14	-2397.970	-2397.854	0.373	-1.603	-1.596	0.007
Pt2Sn6O4	-2335.879	-2335.897	-0.060	-1.027	-1.037	-0.010
Pt2Sn6O6	-2351.327	-2351.438	-0.365	-1.521	-1.542	-0.021
Pt2Sn6O8	-2363.951	-2363.948	0.008	-1.662	-1.669	-0.007
Pt3Sn5O0	-2310.301	-2310.413	-0.378	-0.593	-0.621	-0.028
Pt4Sn4O0	-2312.237	-2312.172	0.217	-0.723	-0.736	-0.013
Pt4Sn4O10	-2373.957	-2373.900	0.189	-1.252	-1.266	-0.014
Pt4Sn4O12	-2384.122	-2384.266	-0.466	-1.086	-1.125	-0.039
Pt4Sn4O2	-2323.377	-2323.342	0.117	-0.678	-0.695	-0.017
Pt4Sn4O4	-2335.791	-2335.864	-0.242	-0.793	-0.823	-0.030
Pt4Sn4O6	-2349.559	-2349.458	0.333	-1.077	-1.085	-0.009
Pt4Sn4O8	-2361.052	-2361.132	-0.263	-1.076	-1.107	-0.031
Pt5Sn3O0	-2311.729	-2311.803	-0.252	-0.548	-0.585	-0.037
Pt6Sn2O0	-2311.416	-2311.358	0.197	-0.397	-0.425	-0.028
Pt6Sn2O10	-2372.425	-2372.122	0.990	-0.837	-0.835	0.003
Pt6Sn2O2	-2324.531	-2324.708	-0.593	-0.599	-0.656	-0.057
Pt6Sn2O4	-2336.156	-2336.570	-1.377	-0.615	-0.702	-0.087
Pt6Sn2O6	-2348.347	-2348.401	-0.178	-0.702	-0.744	-0.042
Pt6Sn2O8	-2359.022	-2359.151	-0.426	-0.599	-0.650	-0.051
Pt7Sn1O0	-2310.394	-2310.584	-0.640	-0.158	-0.223	-0.066
Pt8Sn0O0	-2310.025	-2309.636	1.316	0.000	0.000	0.000
Pt8Sn0O2	-2323.014	-2323.273	-0.867	-0.187	-0.268	-0.081
Pt8Sn0O4	-2335.111	-2335.308	-0.660	-0.261	-0.335	-0.073
Pt8Sn0O6	-2348.855	-2349.070	-0.713	-0.542	-0.618	-0.076
Pt8Sn0O8	-2360.321	-2360.359	-0.125	-0.538	-0.592	-0.053
**RMSE**	**--**	**--**	**0.503**	**--**	**--**	**0.030**
