# Supplementary material for: Zeolite-confined subnanometric PtSn mimicking mortise-and-tenon joinery for catalytic propane dehydrogenation
Source: Nat Commun. 2022 May 17;13:2716. doi: 10.1038/s41467-022-30522-1 (PMC9114386; doi:10.1038/s41467-022-30522-1)
Supplement: Supplementary file 3 — Description of Additional Supplementary Files [file 41467_2022_30522_MOESM3_ESM.pdf]

Description of Additional Supplementary Files

File Name: Supplementary Movie 1
Description: The 2ns MD trajectories of Pt_6 cluster.

File Name: Supplementary Movie 2
Description: The MD trajectories of Sn_6O_6 cluster splitting to Sn_2O_2 and Sn_4O_4 clusters.

File Name: Supplementary Movie 3
Description: The MD trajectories of Sn_8O_8 cluster splitting to Sn_2O_2 and Sn_6O_6 clusters.

File Name: Supplementary Movie 4
Description: The MD trajectories of $Sn_{10}O_{10}$ cluster splitting to Sn_4O_4 and Sn_6O_6 clusters.
